# High throughput Luminex beads based multiplex assay for identification of six major bacterial pathogens of mastitis in dairy animals

**DOI:** 10.3389/fcimb.2023.1125562

**Published:** 2023-07-18

**Authors:** Garima Shrinet, Rajesh Chhabra, Archana Sharma, Kanisht Batra, Saurabh Jyoti Talukdar, Sushila Maan

**Affiliations:** ^1^ Department of Veterinary Microbiology, Lala Lajpat Rai University of Veterinary and Animal Sciences, Hisar, Haryana, India; ^2^ College Central Laboratory, Lala Lajpat Rai University of Veterinary and Animal Sciences, Hisar, Haryana, India; ^3^ Department of Animal Biotechnology, Lala Lajpat Rai University of Veterinary and Animal Sciences, Hisar, Haryana, India

**Keywords:** Luminex, mastitis, bovine, bacteria, high throughput, diagnosis, dairy

## Abstract

**Introduction:**

Bovine mastitis is caused by over 150 different microorganisms. Specific identification and quantification of multiple bacteria in a single milk sample becomes essential for rapid intervention.

**Methods:**

In the present study a Luminex beads based multiplex assay emphasizing on the precise identification of six major bacterial pathogens of mastitis was developed. Assay was developed in two triplex sets, triplex 1 comprised of *Streptococcus agalactiae*, *Streptococcus dysgalactiae* and *Streptococcus uberis* while triplex 2 consisted of *Staphylococcus aureus*, *E. coli* and *Klebsiella pneumoniae*.

**Results:**

The analytical sensitivity was 10 6 copies per reaction mixture for all the six bacteria. A 100% analytical specificity was observed for simultaneous detection of these bacteria. Clinical milk samples from 100 bovine quarters were tested for validation.

**Discussion:**

The analytical sensitivity was similar to the findings reported earlier in real time PCR multiplex assay targeting the DNA of the 11 most common bacterial species or groups in mastitis. The analytical specificity of the optimized assay was 100% similar to reported earlier for simultaneous detection of Mycoplasma spp. and for seven entric viruses of humans.The developed assay indicates a concept proof of a rapid, cost effective high throughput diagnostic tool for identification of major bacteria causing mastitis.

## 1 Introduction

Bovine mastitis is the most prevalent disease in dairy industry characterized by inflammation of the mammary glands; it is severely affecting dairy farms worldwide. The economic losses account due to reduced milk yield, production of low quality milk, cost of drugs and veterinary services, increased culling rate and reduced reproductive efficiency (Down et al., 2017). The economic loss in India alone is estimated to be 1390 INR per lactation due to bovine mastitis (Sinha et al., 2014). In addition, the extensive use of antibiotics in the treatment and control of mastitis have possible implications on human health too. This is through an increased risk of antibiotic resistant strains of bacteria emerging that may enter the food chain (White and McDermott, 2001). The high prevalence of antimicrobial resistance (AMR) in mastitis pathogens has been recorded in different dairy farms suggesting that bovine mastitis potentially jeopardizes both antimicrobial efficacy and public health (Chandrasekaran et al., 2014; Cheng et al., 2019; Abdi et al., 2021; Pascu et al., 2022).

Mastitis is a multi-etiological disease generally represented by co-infection or mixed infection. The etiology of mastitis consists of a wide spectrum of pathogenic agents that penetrate the teat canal and multiply within the udder cistern, produce toxins causing tissue damage. The majority of mastitis cases are produced by a relatively small group of bacteria, comprising of *Staphylococcus aureus*, *Streptococcus agalactiae, Streptococcus dysgalactiae, Streptococcus uberis, E. coli* and *Klebsiella*spp *(* Shome et al., 2011) Therefore, for designing a mastitis prevention and control program, it is worthy to take into account not only the management practices but also the specific identification of the etiological agent and the herd level prevalence of contagious mastitis pathogens.

The gold standard test for mastitis is milk culture for isolation and identification of bacteria, it can take time anywhere between 5-7 days for specific species identification (Dingwell et al., 2003).There can also be possibility of missing out colonies having similar phenotype to the predominant ones (Bradley et al., 2007). Also in more than 26.5% of milk samples from clinical and subclinical bovine mastitis, bacteria fail to grow even after 48 hours on conventional culture (Bradley et al., 2007). Therefore, multiplex or simultaneous identification as well as quantification of major bacteria associated with mastitis in large number of milk samples in one go is desired.

The development of PCR-based methods provided a promising option for the rapid identification of bacteria from mastitis cases (Phuektes et al., 2001; Shome et al., 2011; Ashraf et al., 2017; Calonzi et al., 2020).The disadvantages include high sensitivity that may lead to false positive, minor contaminants in samples leading to misdiagnosis (Phuektes et al., 2001), require agarose gel electrophoresis for interpretation and large number of samples cannot be processed at the same time (Christopher et al., 2013). Above all the number of pathogen identified in multiplex PCR or real time PCR is limited which is challenging considering the diverse list of pathogens of mastitis (Shome et al., 2011).

Consequently, a Luminex beads based multiplex assay designed using xMAP technology reduces time, sample volume, has flexible multiplexing up to 500 targets, allowing high throughput testing and is also a quantitative assay (Deregt et al., 2006; Reslova et al., 2017).It can prove efficient in evaluation of mixed infections like mastitis. Currently very few assays are commercially available for veterinary applications using beads based multiplex assay among which very few are nucleic acid based for detection of pathogens of a disease (Deregt et al., 2006; LeBlanc et al., 2009; Righter et al., 2011; Reslova et al., 2017).Generally most of the Luminex based assays are immunoassays to identify antigens, proteins, antibody productions and differentiation of vaccinated animals from infected (Woolhouse and Alex, 2001; Hsu et al., 2005; Hsu et al., 2007; Watson et al., 2009; Ros et al., 2012).

We here report, development of a nucleic acid based Luminex multiplex assay for qualitative identification of six major bacteria causing mastitis in dairy animals namely *Staphylococcus aureus*, *Streptococcus agalactiae, Streptococcus dysgalactiae, Streptococcus uberis, E. coli* and *Klebsiella pneumoniae.*

## 2 Material and methods

### 2.1 Bacterial strains

ATCC strains of the six bacteria were used as standards for optimization of the assay. The panel included *Staphylococcus aureus* ATCC 12600, *Streptococcus agalactiae* ATCC 12386, *Streptococcus dysgalactiae* ATCC 12394, *Streptococcus uberis* ATCC 700407, *Klebsiella pneumoniae* ATCC 1706 and *E. coli* ATCC 10536 strains. These strains were cultured on 5% sheep blood agar plates at 37°C. The strains were also stored in 30% glycerol stocks as well as lyophilized for further use.

### 2.2 Designing and modification of primers and probes

Published primers sequences targeting the 23S rRNA gene of *Staphylococcus aureus*, 16S rRNA gene of *Streptococcus agalactiae* and *Streptococcus dysgalactiae*, 16-23SrRNA partial sequence of *Klebsiella pnuemoniae*, *Cpn* gene of *Streptococcus uberis* and *PhoA* gene of *E. coli* that are highly conserved genes in the bacteria were selected (Shome et al., 2011). The 5’ Biotin modification of HPLC purified reverse primers depending on strand complimentary to the probe sequence and the PAGE purified forward primers at 50 nmole concentration prepared by Integrated DNA Technology (Coralville, LA, USA) were used.

Specific capture probes that were complementary to organism specific sequences available in Genbank (Accession no.: X68425.1, DQ232512.1, AB002488.1, AF485804.1, FJ546461.1 and DQ399570.1) were designed using BioEdit software version 7.2. The probes had 5’ amino C12 spacer modification and HPLC purification grade. The primer and probe sequence used are listed in **Table 1**.

**Table 1:** The Primer and probe sequences designed for the developed assay.

Target bacteria (gene)	Forward primer (5’-3’)	Reverse primer(5’-3’)	Size (bp)	Probe sequence(5’-3’)
*Staphylococcus aureus* (23S rRNA)	AGCGAGTCTGAATAGGGCGTTT	CCCATCACAGCTCAGCCTTAAC	894	CACTGAATGGAGGACCGAACCGACTTAC
*Streptococcus agalactiae* (16S rRNA)	GCTAATACCGCATAAGAGTAATTAAC	GGTAGATTTTCCACTCCTACCAA	317	CAATTGCTTCACTGTGAGATGGACC
*Streptococcus dysgalactiae* (16S rRNA)	GGGAGTGGAAAATCCACCAT	AAGGGAAAGCCTATCTCTAGACC	572	CTAACGCATTAAGCACTCCGCCT
*Streptococcus uberis* (Cpn)	TCGCGGTATTGAAAAAGCAACAT	TGCAATAATGAGAAGGGGACGAC	400	CAATTTGACCGCGGATACTTATCAC
*E. coli* (Pho A)	GGTAACGTTTCTACCGCAGAGTTG	CAGGGTTGGTACACTGTCATTACG	468	CACATGTGACCTCGCGCAAATGCTAC
*Klebsiella pneumoniae* (16S-23S rRNA)	ATTTGAAGAGGTTGCAAACGAT	TTCACTCTGAAGTTTTCTTGTGTTC	130	CCCGCATAGCTCCACCATCTTTAC

### 2.3 Bacterial genomic DNA extraction from milk samples

The bacterial genomic DNA (gDNA) was extracted from 100 μL of milk tested to be sterile by culturing on LB broth. It was spiked with 10^8^ CFU/mL of ATCC strains of the six bacteria for optimization of the developed assay. Milk Bacterial DNA Isolation Kit (Norgen Biotek Corp., ON, Canada) was used according to the protocol for unknown strain of bacteria. The dsDNA concentration and purity of all six bacterial DNA were measured using the Scandrop^2^ (Analytik Jena, Jena · Germany). Each bacterial species was confirmed by amplification using the primers listed in **Table 1** and visualization in 1.5% agarose gel.

### 2.4 Optimization of multiplex PCR

The multiplex PCR assay was optimized in two triplex combinations. The triplex 1 consisted of *Streptococcus agalactiae, Streptococcus dysgalactiae* and *Streptococcus uberis.* While the triplex 2 comprised of *Staphylococcus aureus, E. coli* and *Klebsiella pneumoniae.*

The conditions for multiplex PCR were optimized through gradient PCR (data not shown). The cycling parameters for triplex 1 consisted of hot start at 95°C for 10 mins, an initial denaturation step of 95°C for 7 mins followed by 30 cycles of 95°C for 30 secs, 58°C for 46 secs, and 72°C for 46 secs with a final extension at 72°C for 10 mins. The cycling parameter for triplex 2 was hot start at 95°C for 10 mins, initial denaturation step of 95°C for 10 mins, followed by 30 cycles of 95°C for 30 secs, 57°C for 45 secs, and 72°C for 45 secs with a final extension at 72°C for 10 mins.

### 2.5 Bead coupling and count

Probes were bound to three different spectrally unique fluorescent beads for both the triplex 1 and 2. The bead coupling protocol and bead count by hemocytometer was conducted as per xMAP cookbook (Angeloni et al., 2013). The bead concentration used was 12.5 million/mL. The bead volume was optimized at 1.25 million beads per specific probe (10 pmole) that was three times lesser than the recommended concentration of beads.

### 2.6 Probe hybridization

Biotinylated PCR products were hybridized to the probe coupled beads in 1.5 X TMACsolution. A total reaction volume of 50 μL comprising of 2.5 μL of the biotinylated PCR product with 33 μL of the working 1.5 X TMAC solution, 1 μL of each labelled beads of the specific triplex set and 11.5 μL of nuclease free water was optimized as working mixture of the assay. Three different hybridization temperatures (50°C, 52°C and 55°C) and time (10, 15 and 30 mins) were tested for both the triplex sets. The detection dye, streptavidin-phycoerythrin (SAPE) was also optimized at 1 μL in 1000 μL (Sigma, 1mg/mL).

### 2.7 Assessment of analytical sensitivity and specificity of assay

The assay reactions were first optimized in the monoplex format using tenfold serial dilutions for each bacteria followed by the triplex format. The wash protocol for hybridization was used. The results were observed on the Luminex xPONENT software version 4.3 and expressed in term of MFI (Mean fluorescence intensity). The limit of detection (LOD) was expressed in terms of copy number. The live pathogenic bacteria were used for calculation of copy number in the defined concentration such as *Streptococcus agalactiae* (7 X 10^8^ CFU/mL), *Streptococcus dysgalactiae* (5.7 X 10^8^ CFU/mL) and *Streptococcus uberis* (9 X 10^8^ CFU/mL), *Staphylococcus aureus* (6 X 10^8^ CFU/mL), *E. coli* (7 X 10^8^ CFU/mL) and *Klebsiella pneumoniae* (5 X 10^8^ CFU/mL). The CFU is indicative of pathogenic load of bacteria with the genomic number present in the sample. The copy number was calculated using the quantified DNA concentration according to the following formulae for copy number = A X N_o_/Length in base pair X 10^9^ X 650. Where A is the DNA concentration in ng/μL, N_o_ is Avogadro’s number (6.022 X 10^23^) and Length is amplicon size.

The specificity of the assay was assessed using the gDNA from the standard ATCC strains of the targeted six bacteria. A negative control consisting of labelled beads with nuclease free water and SAPE was also included. The MFI of the bead coupled probes and biotin labelled PCR product was recorded. Furthermore an *in silico* comparison for cross reactivity of the primers and probe sequences with closely related species to the targeted bacteria was also done using biological sequence alignment editor (BioEdit 7.2) and Basic Local Alignment Search Tool (BLAST).

### 2.8 Repeatability of the assay

The intra and inter assay repeatability were determined. Three separate runs were performed to determine the intra-assay repeatability with 2 replicates and 5 separate runs were performed to determine inter-assay repeatability as per the Luminex xMAP cookbook (Basile et al., 2010).The acceptable range of coefficient of variance (CV) for intra assay repeatability is < 10% and inter assay repeatability is < 20% (Phuektes et al., 2001).

### 2.9 Validation of the assay

To preliminary validate the standardized assay it was tested on bovine milk samples from 40 quarters of suspected cases of mastitis brought to the College Central Laboratory, Lala Lajpat Rai University of Veterinary and Animal Sciences, Hisar, India. Each milk sample was also cultured on blood agar and MacConkey Lactose agar (MLA) simultaneously, to isolate and identify bacteria by conventional culture method and PCR.

## 3 Results

### 3.1 Optimized assay reaction volume, conditions and analysis

The hybridization conditions for triplex 1 were optimized as denaturation at 96°C for 2 mins and annealing at 55°C for 30 mins. While the hybridization condition for triplex 2 was optimized at 98°C for 5 mins and 50°C for 30 mins. The reporter mix was incubated at 50°C for 5 mins and analyzed by the Magpix multiplex reader. The results were observed on the Luminex xPONENT software version 4.3 (**Figures S1, S2**). A low MFI was observed at lower dilution which then became constant on further dilution for *Staphylococcus aureus* and *E. coli* in triplex 2.

### 3.2 Analytical specificity of the assay

**Figure 1:**
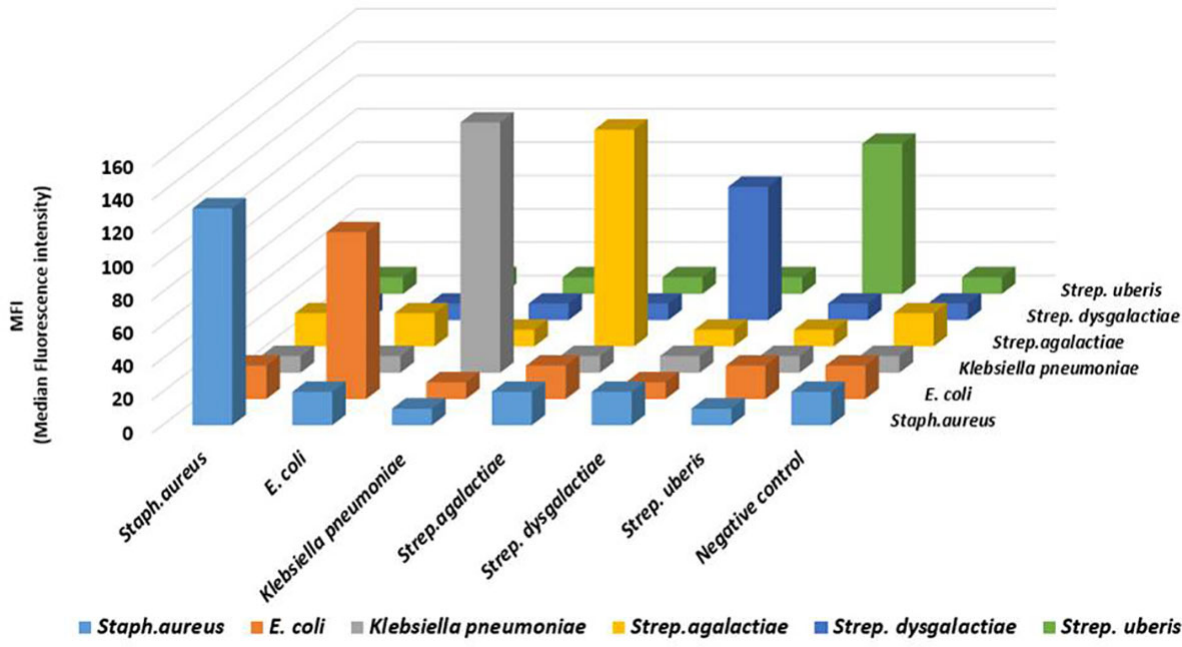
The analytical specificity result of the developed Luminex bead based multiplex assay for bovine mastitis causing bacteria: Analysis of the specificity was carried out for *Staphylococcus aureus*, *Klebsiella pneumoniae*, *E. coli*, *Streptococcus agalactiae*, *Streptococcus dysgalactiae*, and *Streptococcus uberis.* Biotin-labelled PCR products were separated by probe-coupled beads and are presented in terms of dye signal median fluorescence intensity (MFI) in arbitrary units on the y axis. Each peak was identified by beads coupled with specific capture probes and is indicated on the z axis.

**Figure 2:**
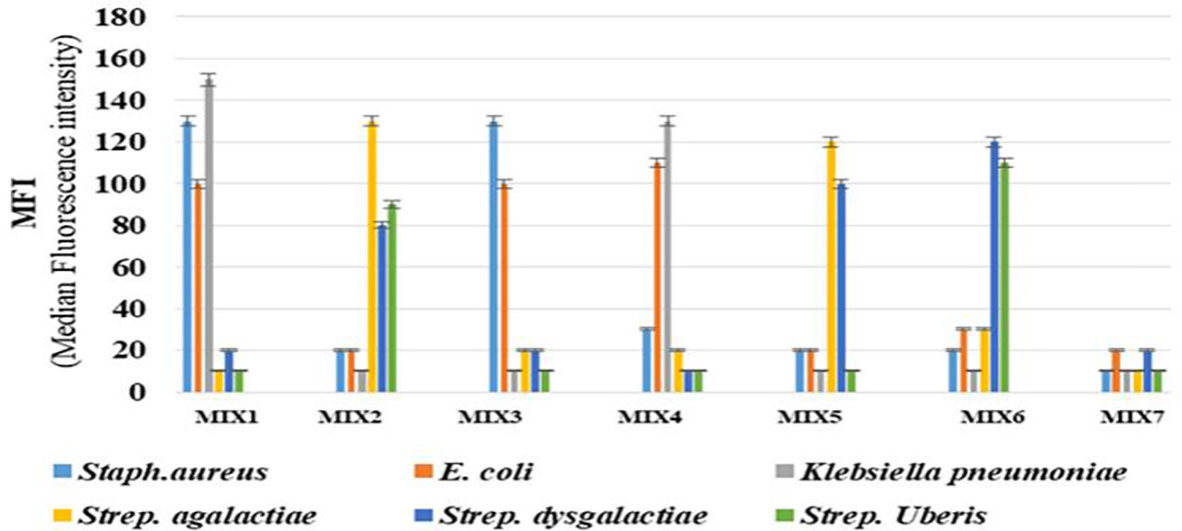
The analysis of mixed-sample detection capacity of the developed Luminex bead based multiplex assay for bovine mastitis causing bacteria: The mixed sample detection capacity of Luminex bead based multiplex assay was carried out using six sets of randomly mixed samples and negative control. Error bars represent 5% of the MFI value. Mix 1: *Staphylococcus aureus, E. coli* &*Klebsiella pneumoniae;* Mix 2: *Streptococcus agalactiae, Streptococcus dysgalactiae*& *Streptococcus uberis;* Mix 3: *Staphylococcus aureus* & *E. coli*; Mix 4: *E. coli* &*Klebsiella pneumoniae;* Mix 5: *Streptococcus agalactia*&, *Streptococcus dysgalactiae;* Mix 6: *Streptococcus dysgalactiae* & *Streptococcus uberis* and Mix 7 or Negative control: Bead + NFW + SAPE.

In this assay, no cross-reactivity with the other four pathogens was found for any of the probes. Also no dye signal corresponding to negative control was seen (**Figure 1**). In order to further assess the performance of the developed assay it was also tested in different target combinations. For this six sets of mixed samples similar to the cases of natural infection and a negative control were included. This showed high specificity of the developed assay for detection of the target with an error bar at 5% (**Figure 2**).

### 3.3 Analytical sensitivity of the assay

The limiting dilution was 10^-6^ for each bacterium in both the triplex set (**Figures S1, S2**). The LOD of each bacterium in triplex 1 and triplex 2 formats was 10^6^ copies per reaction as shown in **Supplementary Tables 1-3**, respectively.

### 3.4 Repeatability of the assay

The CV for each target bacteria in both triplex within a run ranged from 4-7% and 6-9% whereas that between runs ranged from 7-11% and 8-12% for triplex 1 and 2, respectively (**Supplementary Tables 4, 5**).

### 3.5 Validation of the assay on clinical milk samples

To initiate preliminary validation, clinical milk sample from 100 bovine quarters were used. The result showed that analytical sensitivity of the assay was 100% for *Staphylococcus aureus*, *Streptococcus agalactiae,Streptococcus uberis, E. coli* and *Klebsiella pneumoniae.*However, among 7 isolates tested for*Streptococcus dysgalactiae* using culture method and luminex based assay,a single isolateremains unidentified in culture method but identified using luminex based assay. The species identification results by the different methods are given in **Table 2**. The results suggest no significant difference in species identification by the developed assay and PCR, whereas the identification results of the developed assay and conventional culture method showed slight difference in *Streptococcus dysgalactiae* species.

**Table 2:** Identification results of the developed assay in clinical milk samples.

Triplex set	Bacteria species	Positive in Luminex bead based multiplex assay	Positive in PCR	Positive in Culture
Triplex 1	*Streptococcus agalactiae*	2	2	2
	*Streptococcus dysgalactiae*	7	7	6
	*Streptococcus uberis*	3	3	3
Triplex 2	*Staphylococcus aureus*	12	12	12
	*E. coli*	8	8	8
	*Klebsiella pneumonia*	5	5	5

## 4 Discussion

Mastitis is a complex multi- etiological disease of mammary gland, affecting severely the dairy and animal husbandry industry worldwide. It also leads to reservoir of infection for human beings and increased antimicrobial resistance in treated animals (Cheng et al., 2019). Accurate identification of bacterial species is essential to enable successful management strategies, rapid intervention with the use of appropriate treatment and prevention of chronic cases. It is also essential for mastitis pathogen research (Chandrasekaran et al., 2014). Moreover, the “no-growth”samples are problematic for mastitis laboratories, veterinarians, and dairy producers (Shome et al., 2011). This may be due to less number of bacteria in milk, not growing in standard media or substances in milk that inhibit their growth (Gangwal et al., 2017). Reliable identification methods that are fast and accurate still remain a necessity. Recently in the past, various detection methods including PCR, multiplex PCR and real time multiplex PCR have been developed for identification of mastitis causing bacteria (Phuektes et al., 2001; Ashraf et al., 2017; Calonzi et al., 2020). These multiplexed molecular assays are subject to concerns like incompatible or improper amplification, decreased sensitivity and specificity related to nonspecific amplification, incompatible or cross reacting primer sets, high background or noise and poor reproducibility (Koskinen et al., 2009). The earlier studies involving development of a multiplex PCR-coupled Luminex bead based multiplex assay suggested that the microbial detection can be highly sensitive and specific and it is an efficient method for screening multiple pathogens in a single assay and exceeded capacity of real-time PCR ().

The present investigation emphasized on the precise identification of six bacterial pathogens of mastitis in a single sample since it is generally a mixed infection. The protocol can be made high throughput with test of 96 extracted samples in few hours (Angeloni et al., 2013). Thus, the assay was optimized using DNA of known concentration (ng/μL) from ATCC strains of the six bacteria associated with mastitis in two triplex sets; triplex 1 comprised of *Streptococcus agalactiae, Streptococcus dysgalactiae and Streptococcus uberis* whereas, triplex 2 comprised of *Staphylococcus aureus, E. coli* and *Klebsiella pneumoniae.* The Luminex magnetic beads of different spectral address were coupled to the sequence specific probe using the bead coupling protocol of xMAP cookbook, 2013 with some modifications. This coupling reaction was optimized using just 5 million beads that is less than the recommended volume thus, reducing the effective cost of the assay (Capurro et al., 2009).

The analytical sensitivity (LOD) in terms of copy number per reaction was calculated similar to the study of detection of seven enteric viruses (Bruse et al., 2008). That was found to be 10^6^ per reaction for all the six bacteria in monoplex and triplex assays. This was similar to that recorded in the study for detection of different *Mycoplasma* spp. where no difference in LOD was seen in monoplex and multiplex assay (Righter et al., 2011).

A reduced MFI at lower dilution of *Staphylococcus aureus* and *E. coli* were observed which upon further dilution increased and then became constant. This could be explained by the ‘matrix effect’. This implicates to the fact that concentrated complex biological samples such as serum, plasma, or tissue lysates can lead to matrix effect leading to interference or microsphere agglutination, poor bead recovery, low signals and variable results (Angeloni et al., 2013). Moreover the biological range of each analyte, the binding specificity of assay reagents and the unique makeup of the sample must also be known. Dilution of the sample and adding an additional washing step before hybridization can eliminate the matrix effect interference in the results (Christopher et al., 2013). Therefore, in the study washed hybridization protocol and dilution of analyte was performed.

No cross reactivity was observed similar to 100% analytical specificity reported in the assay for simultaneous detection of *Mycoplasma* spp (Righter et al., 2011). as well as in the study for identification of seven enteric viruses of humans (Bruse et al., 2008). The *in silico* comparison of the primer and probe sequence sets with closely related species that are also associated with mastitis showed 100% specificity of the sets with the specific bacterial pathogen similar to finding reported in other study (Taponen et al., 2009).

The performance of the assay was also tested by determining the mixed sample detection capacity of the bead based multiplex assay for both triplex 1 and 2. This was important as in natural infection multiple combination of bacterial pathogens are involved hence it may become difficult to detect. It was found that each peak was identified by beads coupled with specific capture probes. The repeatability of the assay was also assessed in that inter and intra assay coefficient of variance was found within the acceptable range as per Luminex xMAP cookbook (Angeloni et al., 2013).

Any diagnostic assay needs to address whether the assay is “fit for purpose” as described in the validation workflow from the World Organization for Animal Health (OIE) Terrestrial Manual, 2012.

Hence, the preliminary validation of the developed assay was done by evaluation of clinical milk samples from suspected cases of mastitis. The result showed that the percent agreement for identificationwas 100% for *Staphylococcus aureus*, *Streptococcus uberis, E. coli* and *Klebsiella pneumoniae* between developed assay, culture method and PCR. A single isolate of *Streptococcus dysgalactiae* was unidentified in conventional method probably because of missing out the colony due to phenotypical similarity between various *Streptococcus* spp. involved in mastitis (Koskinen et al., 2009).

The concordance between the developed assay and conventional PCR results for all bacteria was 100% that agreed with those recorded earlier for simultaneous detection of enteric viruses (Bruse et al., 2008). The developed assay agreed with the conventional method of bacterial identification from milk by 92.30%, making the assay more sensitive. Though the developed assay had the limitation of detection of only six bacteria in two different sets it could be easily expanded to six-plex assay depending on the availability of beads as the study of cross reactivity of six primers and probes have been done in the present study. Moreover, it is already known that mastitis is caused by 150 different microorganisms, the developed assay can also be further expanded to include more specific probes to identify other bacteria associated with mastitis to the existing platform thus, further reducing the cost of the test.

## Data availability statement

The original contributions presented in the study are included in the article/**Supplementary Material**. Further inquiries can be directed to the corresponding authors.

## Ethics statement

The milk samples used in the study were directly received in the laboratory from the animal owners for bacterial isolation and antibiotic sensitivity testing. History of the animal with the symptoms of mastitis was recorded at time of sample submission. The milking/milk sample collection procedure does not involve invasive procedure therefore ethical permissions are not indicated.

## Author contributions 

GS developed the hypotheses, designed and performed the experiments, analyzed and interpreted data, and drafted the manuscript as a part of his PhD research work; RC administrated the overall research project, oversaw the experiments, analysis and interpretation of data and reviewed the manuscript; AS critically revised the manuscript; KB assisted with the experiments; ST helped in genomic DNA isolation from bacterial colonies and milk samples; SM helped in probe designing and provided expertise on development of Luminex assay and reviewed the manuscript. All authors contributed to the article and approved the submitted version.
