# Supplementary material for: High throughput Luminex beads based multiplex assay for identification of six major bacterial pathogens of mastitis in dairy animals
Source: Front Cell Infect Microbiol. 2023 Jul 18;13:1125562. doi: 10.3389/fcimb.2023.1125562 (PMC10390833; doi:10.3389/fcimb.2023.1125562)
Supplement: Supplementary file 1 [file DataSheet_1.docx]

**Supplementary Material**

**Figures**

Figure S1: Optimized assay for triplex 1 including *Streptococcus agalactiae, Streptococcus dysgalactiae* and *Streptococcus uberis* (10 fold serial dilutions)


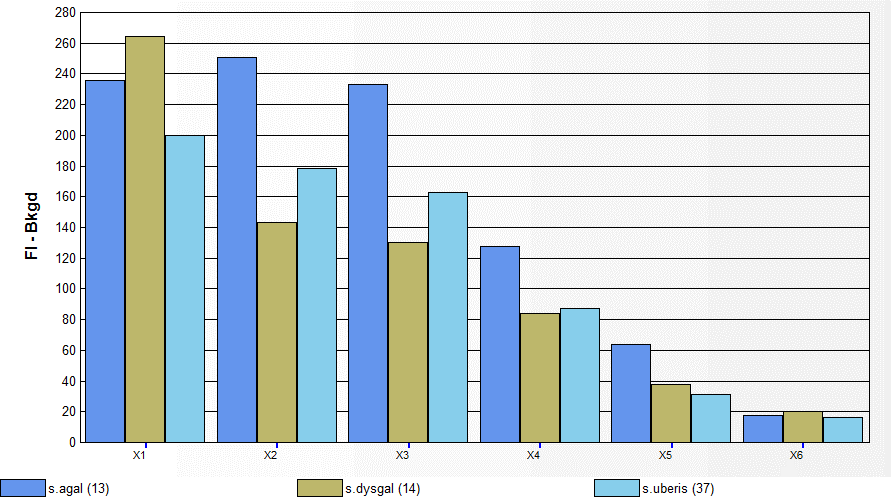


The x axis represents the fluorescence intensity minus the background intensity for respective bacteria, while the y axis represents the 10 fold serial dilution of bacteria used in the triplex assay.

Figure S2: Optimized assay for triplex 2 including *Staphylococcus aureus, Klebsiella pneumoniae* and *E. coli* (10 fold serial dilutions)

**
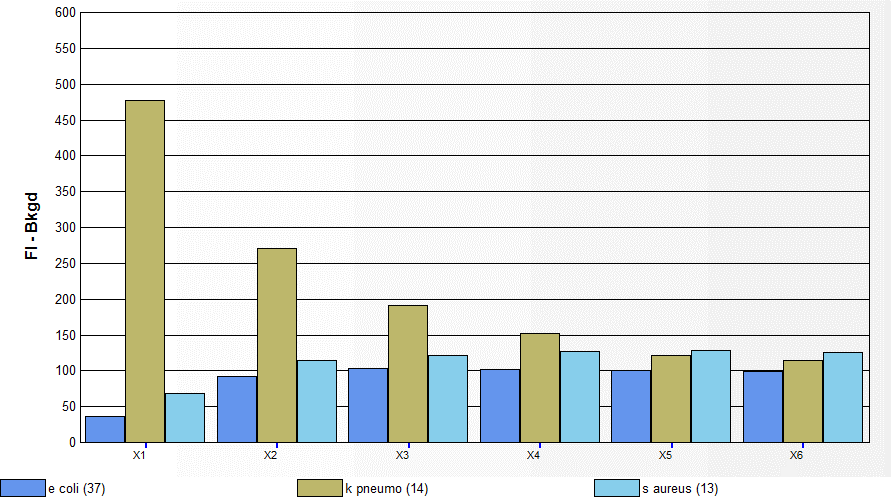
**

The x axis represents the fluorescence intensity minus the background intensity for respective bacteria, while the y axis represents the 10 fold serial dilution of bacteria used in the triplex assay.

**Tables**

**Supplementary table 1: The Limit of detection (LOD) in monoplex format**

| **S No.** | **Bacterial species** | **LOD (Copy number)** |
| --- | --- | --- |
| 1 | *Streptococcus agalactiae* | 4.13 X 10^6^ |
| 2 | *Streptococcus dysgalactiae* | 2.88 X 10^6^ |
| 3 | *Streptococcus uberis* | 3.91 X 10^6^ |
| 4 | *Staphylococcus aureus* | 7.95 X 10^6^ |
| 5 | *E. coli* | 3.68 X 10^6^ |
| 6 | *Klebsiella pneumonia* | 1.23 X 10^6^ |

**Supplementary table 2: The Limit of detection (LOD) in triplex 1**

| **S No.** | **Bacterial species** | **LOD (Copy number)** |
| --- | --- | --- |
| 1 | *Streptococcus agalactiae* | 4.335 X 10^6^ |
| 2 | *Streptococcus dysgalactiae* | 1.909 X10^6^ |
| 3 | *Streptococcus uberis* | 3.2665 X 10^6^ |

**Supplementary table 3: The Limit of detection (LOD) in triplex 2**

| **S No.** | **Bacterial species** | **LOD (Copy number)** |
| --- | --- | --- |
| 1 | *Staphylococcus aureus* | 1.365 X 10^6^ |
| 2 | *E. coli* | 3.073 X 10^6^ |
| 3 | *Klebsiella pneumonia* | 1.066 X 10^6^ |

| **S No.** | **Analyte** | **Inter- assay (CV %)** | **Intra-assay (CV %)** |
| --- | --- | --- | --- |
| 1 | *Streptococcus agalactiae* | 7.4 | 11 |
| 2 | *Strepococcus dysgalactiae* | 4.6 | 9.1 |
| 3 | *Streptococcus uberis* | 4.47 | 7.9 |

**Supplementary table 4: CV % of inter-assay and intra-assay repeatability for triplex 1**

**Supplementary table 5: CV % inter- assay and intra-assay repeatability for triplex 2**

| **S No.** | **Analyte** | **Inter – assay (CV %)** | **Intra – assay (CV %)** |
| --- | --- | --- | --- |
| 1 | *Staphylococcus aureus* | 9.6 | 12.1 |
| 2 | *E. coli* | 6.3 | 8.9 |
| 3 | *Klebsiella pneumoniae* | 3.7 | 5.6 |
